# Coral Reef Water Microbial Communities of Jardines de la Reina, Cuba

**DOI:** 10.3390/microorganisms12091822

**Published:** 2024-09-03

**Authors:** Manon Denux, Maickel Armenteros, Laura Weber, Carolyn A. Miller, Kinga Sántha, Amy Apprill

**Affiliations:** 1 Woods Hole Oceanographic Institution, 266 Woods Hole Road, Woods Hole, MA 02543, USA; denuxmanon@gmail.com (M.D.);; 2 Unidad Académica Mazatlán, Instituto de Ciencias del Mar y Limnología, Universidad Nacional Autónoma de México, Mazatlán 82040, Mexico; 3 Faculty of Geosciences and Environment, University of Lausanne, CH-1015 Lausanne, Switzerland

**Keywords:** coral reef, microorganism, 16S rRNA gene, diversity, marine protected area

## Abstract

Globally, coral reef ecosystems are undergoing significant change related to climate change and anthropogenic activities. Yet, the Cuban archipelago of Jardines de la Reina (JR) has experienced fewer stressors due to its geographical remoteness and high level of conservation. This study examines the surface and benthic reef water microbial communities associated with 32 reef sites along the JR archipelago and explores the relationship between the community composition of reef microorganisms examined using bacterial and archaeal small subunit ribosomal RNA gene (16S rRNA gene) sequencing compared to geographic, conservation/protection level, environmental, physicochemical, and reef benthic and pelagic community features. Reef nutrient concentrations were low and microbial communities dominated by picocyanobacteria and SAR11 and SAR86 clade bacteria, characteristic of an oligotrophic system. Reef water microbial community alpha and beta diversity both varied throughout the archipelago and were strongly related to geography. Three sites in the western archipelago showed unique microbial communities, which may be related to the hydrogeography and influences of the channels linking the Ana Maria gulf with the Caribbean Sea. Overall, this work provides the first extensive description of the reef microbial ecology of the Caribbean’s ‘Crown Jewel’ reef system and a framework to evaluate the influence of ongoing stressors on the reef microorganisms.

## 1. Introduction

Coral reefs are one of the most biodiverse and economically valuable ocean ecosystems. These complex, rich, and productive ecosystems have persisted despite their presence in oligotrophic waters, partially due to their reliance on symbiosis and coupling between macroorganisms and the abundant and diverse assemblies of microorganisms [1]. Coral reefs have undergone significant and recent changes and declines, related to eutrophication [2], overfishing [3], disease outbreaks [4], and climate change induced ocean warming and acidification [5]. Understanding reef ecosystem health has become increasingly challenging due to this shifting baseline and increasing number of stressors. Microorganisms, the most abundant, diverse, and fastest growing and evolving members of the reef ecosystem, have the potential to serve as bioindicators for examining shifting baselines on reefs [6,7].

Microorganisms, including bacteria, archaea, picoeukaryotes, and viruses, are widely associated with reef organisms, sediments, and the overlying reef seawater [8,9]. The reef seawater bacteria and archaea (or picoplankton) are the most extensively studied members of these communities, and their composition is influenced by large-scale biogeographic processes but also smaller-scale ecological processes [10,11,12,13,14]. Studies have documented how specific benthic organisms, including corals, sponges, and macroalgae, influence the composition of the surrounding picoplankton community, with benthic organisms supplying growth substrates or promoters [15,16], as well as some organisms predating on specific picoplankters. For example, the coral *Porites astreoides* exudates enhances the growth of the *Roseobacter* HIMB11 and even oligotrophic taxa such as SAR11 and OMB60 (NOR5) bacteria, and this coral also preys on *Synechococcus* [17,18]. Fish and motile invertebrates also likely contribute to reef water microbial community dynamics, although there are fewer targeted studies documenting specific interactions [19].

The Jardines de la Reina (JR) Cuban archipelago is an ideal environment to examine the impact of reef community composition on water picoplankton without direct human influences. JR is acknowledged as a pristine, ‘crown jewel’ coral reef ecosystem in the Caribbean. It is located offshore southeastern Cuba, and the combination of its remote nature and unique and effective protective status have contributed to its success, with this ecosystem supporting some of the highest fish biomass and coral cover in the Caribbean [20]. There is an absence of large-scale industrialized agriculture or extensive development of Cuba’s coastline, and the JR archipelago is further buffered from the land by the Gulf of Ana Maria which harbors a succession of small mangrove keys and extensive seagrass meadows with corresponding sediments responsible for trapping organic matter and nutrients. On its southern side, the archipelago is flushed by the main surface circulation in the Caribbean Sea, i.e., the Caribbean current which carries warm oligotrophic waters westward. It is the largest marine protected area (MPA) in the Caribbean region (ca. 2000 km^2^), and it shows evidence of enforcement such as having the highest fish biomass of the Caribbean [21]. Additionally, tourism is highly restricted on the reefs, with less than 1000 visitors allowed per year. As coral reef health declines across the Caribbean Sea, JR provides an opportunity to examine the functioning and diversity of a protected and more pristine ecosystem.

In 2015, members of our team examined picoplankton communities associated with four JR reefs [22]. We found that the JR reefs harbored unique picoplankton communities compared to reefs from Gulf of Batabanó, Cuba, and the Florida Keys, USA. Indeed, the JR reef waters showed consistently high microbial diversity with low beta diversity compared to the other regions, suggesting that the pristine and protective nature of JR reefs may be related to these unique characteristics. In 2017, we had the opportunity to return to the JR region under a USA–Cuba cooperative agreement to conduct the first large-scale survey of the microbial ecology of the JR region and examine the consistency of microbial characteristics along the archipelago and possible sources of variability.

For this study, we sampled surface and reef-depth waters across 32 reef sites in the JR archipelago to examine the microbial community composition using partial 16S rRNA gene sequencing targeting bacteria and archaea, in relation to a larger framework of environmental and reef characteristics as well as the geographic position and protective status of each site. We aimed to first investigate the diversity and community structure (i.e., abundance and composition) of microbes across the archipelago. Further, we examined how geography, environmental variables, and protection enforcement levels influence microbial diversity and community structure.

## 2. Materials and Methods

### 2.1. Study Region

**Figure 1:**
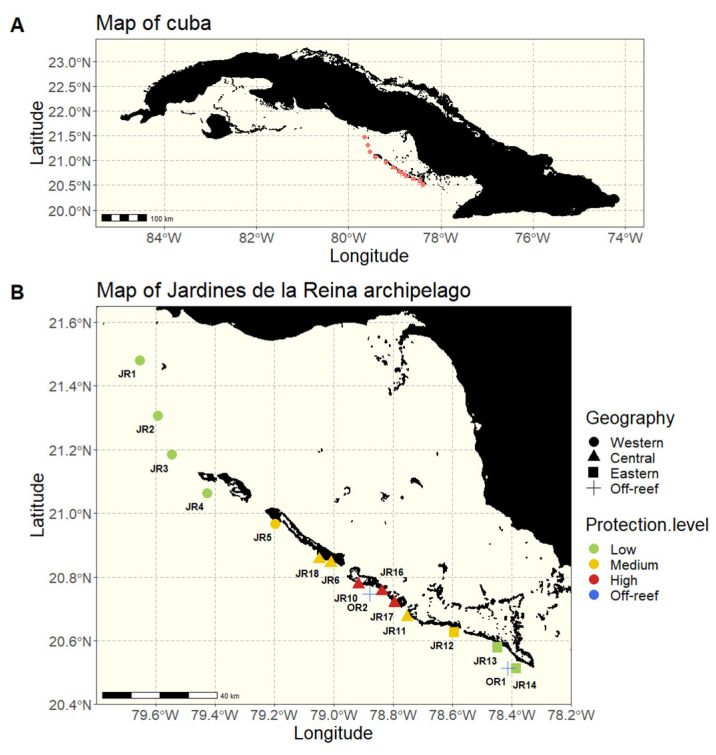
Maps of Cuba and the 16 studied reefs. (**A**) A map of Cuba, the red dots representing the 16 studied reefs. (**B**) A map of the Jardines de la Reina (JR) archipelago with sampled reefs grouped according to their geographical location (western, central, eastern, and off-reef) and colored according to the protection enforcement level (low, medium, high, and off-reef).

We sampled 32 reefs spanning approximately 200 km along the JR archipelago in Cuba during November 2017 (Figure 1). At 16 reefs throughout the archipelago, we sampled two sites (JRa and JRb) to capture variability within each geographic area. The JRa and JRb sites were located 125–1300 m apart with a mean distance of 731 m between a and b sites. The exception is that JR1, OR1, and OR2 only had a JRa site, and JR16 was sampled 3 times (sites a, b, and c). Off-reef waters (OR) were also sampled to provide a comparison to non-reef locations (*n* = 2 off-reef sites). The sites were all on the forereef, either reef terrace or spur and groove, and differed in their geographic location within the archipelago as well as their MPA protection enforcement level established in 1996 (Table 1). Historic protection categories were based on descriptions by Pina-Amargós et al. [23,24] to reflect the unevenness of protection over a large territory due to limited park resources which results in the occurrence of poaching towards the boundaries of the MPA.

### 2.2. Physicochemical Properties of Seawater

A conductivity, temperature, and depth sonde (CTD; YSI Exo Sonde, Xylem Inc., Yellow Springs, OH, USA) was deployed at each site to survey the physicochemical properties (temperature, salinity, dissolved oxygen, and pH) of the water column. A component of these environmental data was previously presented by Weber et al. [25] and Navarro-Martínez et al. [26].

### 2.3. Benthos and Fish Population Surveys

Benthic cover of all reef sites was surveyed by SCUBA divers using the line-intercept survey method [27]. Between 10 and 20 transects (10 m in length) across the reef substrate led to the determination of percent cover of coral, algae, sponges, rocks, and sand. The data from half of the study sites presented here were previously examined by Weber et al. [25]. For the present study, coral, algae, and sponge categories were incorporated into the analyses.

Fish species richness and abundance were examined with 9–12 transects per site, each consisting of a 25 m × 5 m belt transect recorded with a diver-operated stereo-video (stereo-DOV) as described and previously presented by Navarro-Martínez et al. [26]. From this footage, researchers determined fish species richness as the average number of species per study area (the sum of the number of species in every transect divided by the number of transects per study area). Furthermore, fish abundance was determined using the average number of fish specimens per site (the sum of the number of specimens in every transect divided by the number of transects per study area).

Coral species richness and abundance were assessed using photographs at high resolution with a plastic quadrat of 25 cm × 25 cm laid on the bottom as described by Hernández-Fernández et al. [28]. For each reef, 10 replicates were made randomly distributed within a diameter of ca. 50 m from a central point. Coral species richness and abundance were calculated as previously described for fish.

Statistical comparisons of reef and environmental data were conducted using a Kruskal–Wallis rank-sum test followed by a post hoc Dunn’s test with Bonferroni corrections.

### 2.4. Macronutrients, Cell Count, and Pigment Analysis

Samples for inorganic nutrients, total organic carbon and nitrogen, and microbial cell abundance surveys were collected, processed, and previously described by Weber et al. [25]. Seawater was collected from both surface (<1 m depth) and reef depth (~1 m above reef), with depths ranging from 6 to 14 m. For off-reef sites, only surface water was sampled.

Surface and reef-depth seawater samples (40 mL) were collected for organic macronutrient analysis, acidified with 75 µL of concentrated phosphoric acid, capped, and stored at room temperature before analysis on a Shimadzu TOC-VCSH TOC analyzer (Shimadzu Scientific Instruments, Columbia, MD, USA) [29] with a TNM-1 module to measure concentrations of non-purgeable total organic carbon (NPOC also referred as TOC, unfiltered) and total nitrogen (TN, the combination of organic and inorganic nitrogen, unfiltered). Surface and reef-depth seawater samples (30 mL) collected for analyses of inorganic macronutrient concentrations were frozen prior to analysis. Concentrations of inorganic macronutrients including phosphate (PO_4_^3−^), nitrite + nitrate (N + N), nitrite (NO_2_^−^), ammonium (NH_4_^+^), and silicate (SiO_3_^2−^) were measured with a continuous segmented flow system as described by Apprill and Rappé [30]. The detection limits of the instrument varied according to the macronutrient as so: phosphate = 0.01 µM, nitrite + nitrate = 0.07 µM, nitrite = 0.01 µM, and ammonium = 0.02 µM. The concentration of nitrate was calculated by subtracting the concentration of nitrite from the sum of the two (N + N). Total organic nitrogen (TON) was acquired for this study by subtracting the sum of the inorganic nitrogen species (NH4^+^ + (N + N)) from TN.

Unfiltered seawater samples of 1 mL collected for enumeration of *Prochlorococcus* (PRO), *Synechococcus* (SYN), picoeukaryotic cells (PEUK), and unpigmented cells (referred to simply as heterotrophic bacteria and archaea, HBACT, but cellular metabolisms were not determined) were fixed with paraformaldehyde (1% final volume), incubated at 4 °C in the dark for 30 min, and stored at −80 °C prior to analysis. Pigmented and unpigmented cells were run separately for each sample by flow cytometry using the collinear analysis method and a UV wavelength of 488 nm on an Altra flow cytometer at the University of Hawaii. Unpigmented cells were stained with Hoechst stain. Each cell type abundance was estimated by binning populations using FlowJo (v. 6.4.7) software.

For phytoplankton pigment analysis (chlorophyll a and phaeophytin), 2–4 L of reef-depth seawater was filtered through GF/F glass microfiber filters (Whatman^®^, Cytiva, Marlborough, MA, USA). After pigment extraction from the filters using 90% acetone in water, the optical density (OD) values were measured by a calibrated spectrophotometer using standard optics (Lambda 18, Perkin Elmer, Waltham, MA, USA).

Statistical comparisons of reef and environmental data were conducted using a Kruskal–Wallis rank-sum test followed by post hoc Dunn’s test with Bonferroni corrections.

### 2.5. 16S rRNA Gene Sequencing

Surface and reef-depth water samples were collected from each location, and duplicate samples of seawater from each depth were filtered (1–2 L) using 0.2 μm pore size, 25 mm Supor^®^ PES filters (Pall Corporation, Port Washington, NY, USA) under peristaltic pressure. Filters were stored in cryovials, flash-frozen in liquid nitrogen, and stored at −80 °C. DNA was extracted from filters using DNeasy^®^ PowerBiofilm^®^ (Qiagen, Hilden, Germany) using the manufacturer’s protocols. Microbial community composition was assessed by targeting the V4 region of the 16S rRNA gene using 515FY and 806RB primers, as outlined by Parada et al. [31] and Apprill et al. [32] with unique barcoded primer combinations for each sample. Each sample was processed in 50 μL PCR reactions containing 2.5 U of GoTaq Flexi DNA Polymerase (Promega Corporation, Madison, WI, USA), 10 μL 5X Colorless GoTaq Flexi Buffer, 2.5 mM MgCl_2_, 0.2 mM dNTP mix, 0.2 μM of each barcoded primer, and 2 μL of the sample or control. The PCR reactions were carried out on a C1000™ Thermal Cycler from Bio-Rad (Bio-Rad Laboratories, Inc., Hercules, CA, USA) and consisted of an initial denaturation step at 95 °C for 2 min, followed by 24, 26, or 28 cycles of 95 °C for 20 s, 55 °C for 15 s, and 72 °C for 5 min, concluding with an extension at 72 °C for 10 min. PCR products were screened using gel electrophoresis on 1% agarose/tris-borateEDTA gels (120 V for 30 min) stained with SYBR™ Safe gel stain (Invitrogen™, Waltham, MA, USA) and with the HyperLadder™ 50 bp DNA ladder (Bioline, London, UK) as a size reference. PCR cycles varied slightly between samples to ensure that they produced similar minimal PCR product yields across samples. DNA extraction and PCR controls (blanks and positive controls of seawater DNA known to amplify) were used to control for potential contamination from reagents. Moreover, amplification bias and sequencing errors were monitored using genomic DNA from Microbial Mock Community B (even, low concentration), v3.1, HM-782D, obtained through BEI Resources, Manassas, VA, USA; NIAID, NIH as part of the Human Microbiome Project). PCR products were purified using the MinElute^®^ PCR Purification kit (Qiagen, Valencia, CA, USA), and quantified via the Invitrogen™ Quant-iT™ PicoGreen™ dsDNA Assay Kits and dsDNA Reagents (Thermo Fisher Scientific Inc., Waltham, MA, USA). Finally, amplicons were pooled in equimolar ratios and sequenced using the 2 × 250 bp paired-end Illumina MiSeq method (Illumina, San Diego, CA, USA), similar to Kozich et al. [33], at the University of Illinois W.M. Keck Center for Comparative and Functional Genomics.

### 2.6. Sequencing Data Analysis

Demultiplexed sequences were processed using the R package dada2 (v 1.24.0) [34] according to the DADA2 Pipeline Tutorial (v 1.16, https://benjjneb.github.io/dada2/faq.html, accessed on 30 March 2023). The filtering parameter truncLen was set to truncate forward reads at 240 bp and reverse reads at 150 bp, where observed quality scores (QSs) on quality profiles began to drop below 30. All other parameters were kept at default values. Paired reads were merged before sequences were clustered into amplicon sequence variants (ASVs); then, chimeras were removed. By the end of the dada2 processing, between 70.9 and 90.8% of input reads passed quality control in seawater samples, and 80.8–82.1% of input reads passed quality control in the mock community samples (positive control). Taxonomy was assigned using the SILVA SSU rRNA database (v.138.1) down to the species level when possible [35].

Contaminant ASVs were identified and removed from all samples using the prevalence method with a threshold of 0.2 from the decontam (v.1.13.0) R package [36]. This method identified 12 ASV contaminants, with enriched frequency in DNA extraction negative controls. Those 12 ASVs were removed as well as positive and negative control samples, leaving 7698 ASVs for comparison in this study.

Before alpha diversity analysis, a random subsampling was performed to reach 33,781 reads for each sample. This threshold was chosen to preserve the total diversity of the highest number of samples. This rarefaction process aims at normalizing sample sizes to minimize the impacts of uneven sequence coverage across samples. Alpha diversity metrics were then determined using the “estimate_richness()” function from the R package “phyloseq” (v.1.40.0) [37]. To visualize the taxonomic composition of each site, ASV counts were transformed into relative abundances and only ASVs with an average relative abundance across samples greater than 0.01 were plotted.

The R package “corncob” (v.0.3.1) [38] was used to identify ASVs significantly differentially abundant between geographic locations. Corncob was run using the parametric Wald test with an FDR cut-off of 0.05. Two hypotheses were tested: (1) The relative abundance of some ASVs are significantly different according to the reef location across the JR archipelago due to hydrogeography as well as other reef-specific biological and physical properties and (2) sites with atypical alpha and beta diversity features (‘abnormal’ reefs) harboring ASVs that are significantly different in relative abundance from other reefs. The first hypothesis was tested by comparing ASV counts found in the western reefs with the ones found in both the central and eastern reefs, after excluding abnormal reefs (JR11, JR12, and JR14) as well as off-reef sites from the dataset. Such an opposition accounts for the fact that the western reefs are likely more impacted by Ana Maria gulf waters due to the absence of islands acting as physical barriers. The second hypothesis was tested only for reef-depth samples, and therefore, off-reef sites were excluded from the test because only surface water was collected.

A Bray–Curtis dissimilarity matrix was calculated using the “vegdist()” function from R package “vegan” (v.2.6.4) [39]. The beta diversity of communities was assessed as mean distance to the centroid of the group for each geographic locations and protection levels using the “betadisp()” function from “vegan” R package [39]). Note that the number of sequences per sample ranges from 18,409 to 460,146, with a mean (std. dev.) of 75,893 (61,177) sequences. Reef and environmental data were log-transformed to ensure normality. To visualize the microbial community structure, we used nonmetric multidimensional scaling (nMDS) with overlaid environmental variables fit onto the ordination using the “envfit()” function (“vegan” R package [39]). Furthermore, a permutational multivariate ANOVA (PERMANOVA), function “adonis2()” from the “vegan” R Package [39]) was conducted to determine the environmental variables that have a significant (*p* ≤ 0.05) influence on microbial community structure.

## 3. Results

### 3.1. Reef Biogeochemistry

The physicochemical properties of the sites were generally homogeneous across the JR reef system. Seawater temperature ranged between 28.2 and 28.8 °C, dissolved oxygen ranged between 6.02 and 6.67 mg L^−1^, salinity ranged between 36.9 and 37.1, and pH ranged between 8.26 and 8.33. Reef benthic communities were previously presented [25], with coral cover ranging from 11 to 35%, algal cover ranging from 24 to 41%, and around 4% of sponge cover. Benthic cover was generally similar between sites, with few exceptions [25]. Fish community data were also previously presented for these sites and for the same cruise and showed variability in communities along the archipelago [26]. The distance from the center of the MPA was related to some of the variability in the abundance, functional richness, and structure of fish communities [26].

**Figure 2:**
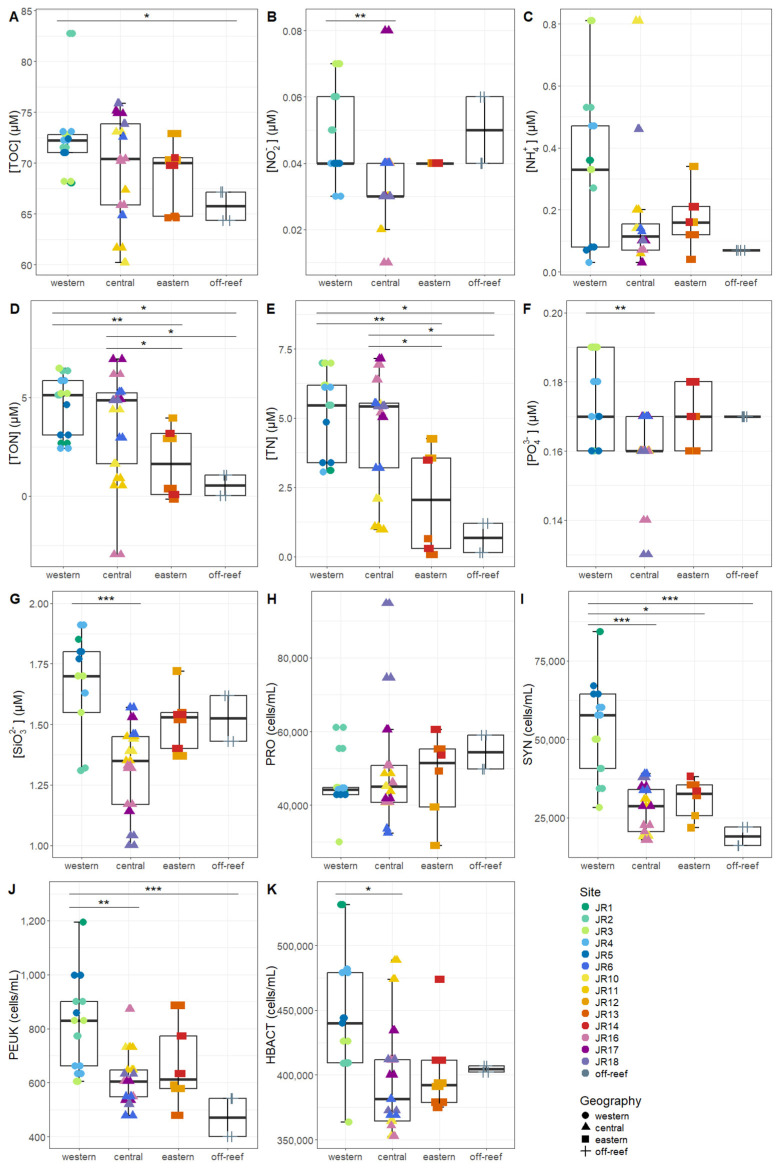
The environmental signatures of surface seawater microbial communities, including (**A**) total organic carbon (TOC), (**B**) nitrite (NO_2_^−^), (**C**) ammonium (NH_4_^+),^ (**D**) total organic nitrogen (TON), (**E**) total nitrogen (TN), (**F**) phosphate (PO_4_^2−^), (**G**) silicate (SiO_3_^2−^), (**H**) *Prochlorococcus* (PRO), (**I**) *Synechococcus* (SYN), (**J**) picoeukaryotes (PEUK), and (**K**) heterotrophic bacteria (HBACT). The colors designate each reef, the horizontal thick black bar across the boxplot represents the median value, and the whiskers extend to values at 1.5 times the interquartile range (delimited by the box). The horizontal black bars at the top and their associated asterisks indicate significantly different groups as identified via the Kruskal–Wallis rank-sum test and Dunn’s test with Bonferroni corrections (*: *p* < 0.05, **: *p* < 0.01, ***: *p* < 0.001).

**Figure 3:**
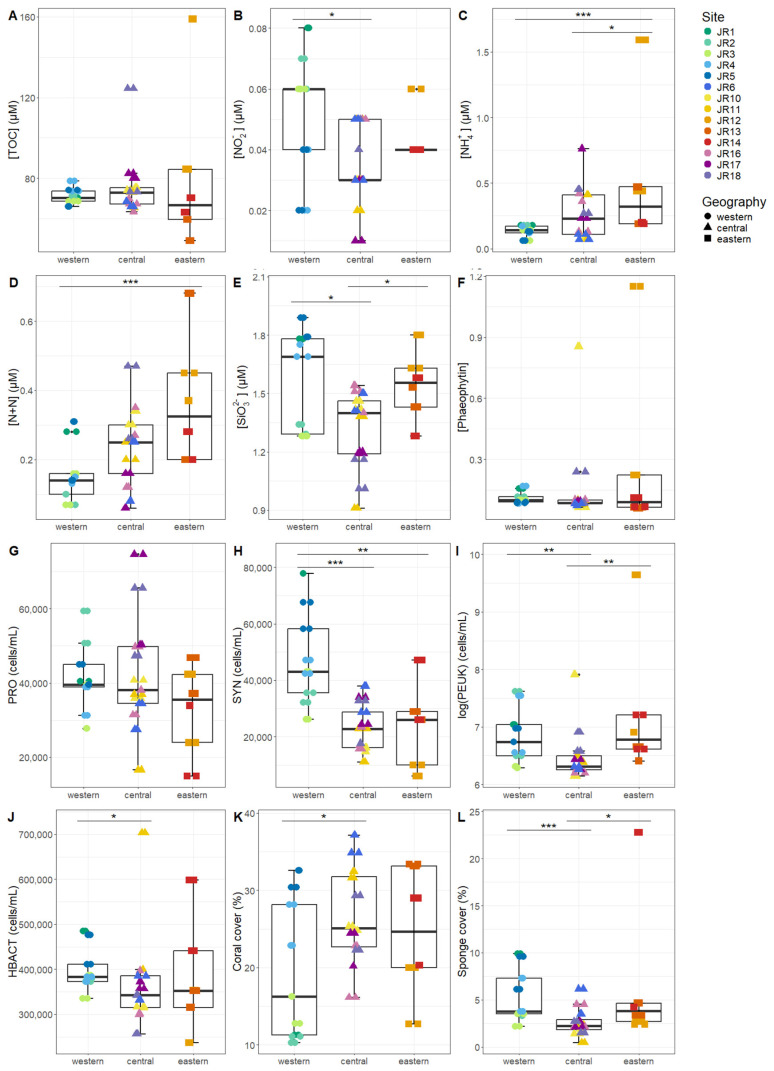
The environmental signatures of reef-depth seawater microbial communities, including (**A**) total organic carbon (TOC), (**B**) nitrite (NO_2_^−^), (**C**) ammonium (NH_4_^+),^ (**D**) nitrite + nitrate (N + N), (**E**) silicate (SiO_3_^2−^), (**F**) phaeophytin, (**G**) *Prochlorococcus* (PRO), (**H**) *Synechococcus* (SYN), (**I**) picoeukaryotes (PEUK), (**J**) heterotrophic bacteria (HBACT), (**K**) coral cover, and (**L**) sponge cover. The colors designate each reef, the horizontal thick black bar across the boxplot represents the median value, and the whiskers extend to values at 1.5 times the interquartile range (delimited by the box). The horizontal black bars at the top and their associated asterisks indicate significantly different groups as identified via the Kruskal–Wallis rank-sum test and Dunn’s test with Bonferroni corrections (*: *p* < 0.05, **: *p* < 0.01, ***: *p* < 0.001).

Macronutrients were generally low at the reef sites, with total nitrogen (TN, the combination of organic and inorganic nitrogen) displaying the widest range, due to elevated values at site JR12 (Table 2). Concentrations of *Synechococcus* and picoeukaryotes were more variable between sites compared to *Prochlorococcus* and unpigmented picoplankton abundances (Table 2). Macronutrients and microbial abundances were examined and compared separately for surface and benthic depths of the reefs (Figure 2 and Figure 3). Off-reef surface waters were significantly lower in TON and TN as well as abundances of *Synechococcus* and picoeukaryotes compared to the reef surface waters. Surface waters of the western reefs showed significantly elevated nutrients (TON, TN, and silicate) as well as *Synechococcus*, picoeukaryote, and heterotrophic cells compared to the other geographic regions (Figure 2). For reef-depth water, nutrient and cell abundances were more similar between geographic locations. The western reefs were associated with lower ammonium and higher abundance of *Synechococcus* and heterotrophic bacteria compared to the other locations (Figure 3).

### 3.2. Microbial Community: Alpha Diversity

Our analyses detected variability in reef water microbial alpha diversity between the reef sites. For microbial species richness, values ranged from 243 to 1225 ASVs (Mean ± SD 444 ± 169 ASVs). For the Shannon index, values ranged from 4.11 to 5.28 (Mean ± SD 4.55 ± 0.24). No significant difference in alpha diversity was found between the surface (<1 m) and reef depths (6–14 m), and therefore, the data were combined for the remaining alpha diversity analyses (Figure S1).

**Figure 4:**
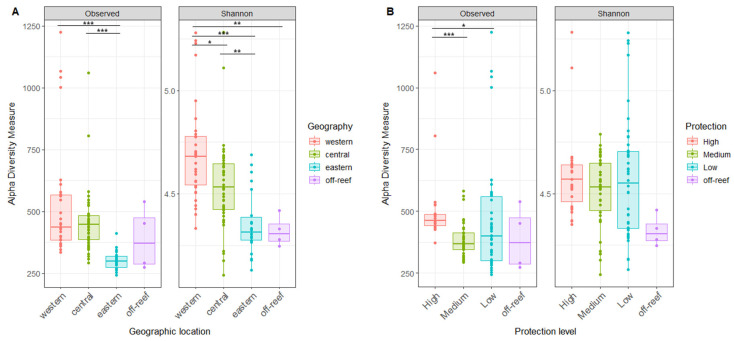
The microbial alpha diversity of reef waters differs by geography and reef protection levels. (**A**) The richness and Shannon index of reef water compared by geographic locations. (**B**) The richness and Shannon index of reef water compared by protection levels. The horizontal thick bar across the boxplot represents the median value, and the whiskers extend to values at 1.5 times the interquartile range (delimited by the box). The horizontal black bars at the top and their associated asterisks indicate significantly different groups as identified via the Kruskal–Wallis rank-sum test and Dunn’s test with Bonferroni corrections (*: *p* ≤ 0.05, **: *p* ≤ 0.01, ***: *p* ≤ 0.001).

Alpha diversity differed according to geographic location, with the highest alpha diversity in the west, followed by the central and then eastern reefs, with significant differences in the Shannon index between all reef regions (Figure 4A). The off-reef site Shannon index site was significantly lower than the western reefs (Figure 4A). Alpha diversity also differed by the level of reef protection, with the highest microbial richness in most protected reefs, but this trend was not confirmed by the Shannon Index (Figure 4B).

### 3.3. Microbial Community: Beta Diversity

**Figure 5:**
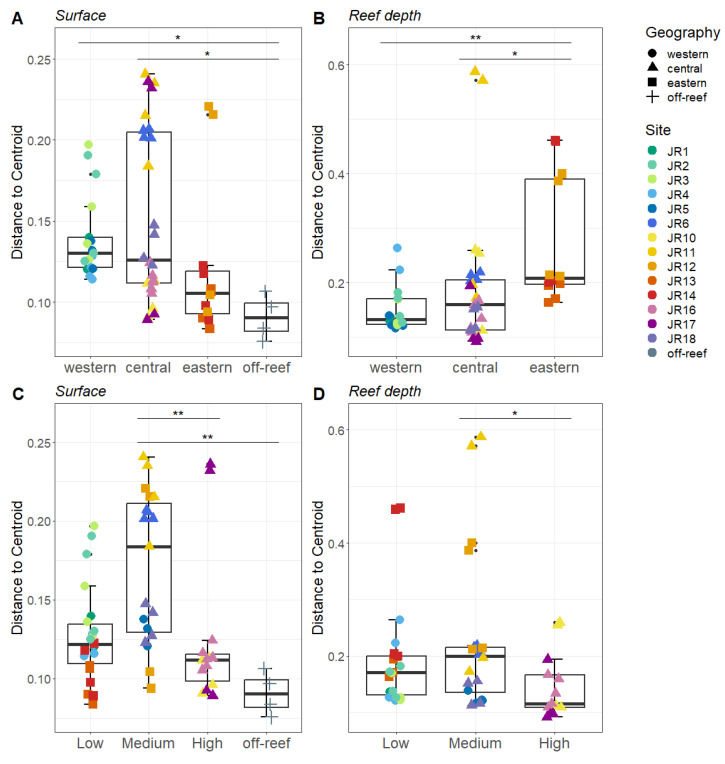
Beta diversity measured as the average distance of reefs to the group centroid. The reefs are divided between surface ((**A**,**C**) depth < 1 m) and reef depths ((**B**,**D**) depth = 6–14 m). The reefs are grouped by geographical locations (**A**,**B**) and by protection levels (**C**,**D**). The colors designate each reef, the horizontal thick black bar across the boxplot represents the median, and the whiskers extend to 1.5 times the interquartile range (delimited by the box). The horizontal black bars at the top and their associated asterisks indicate significantly different groups as identified via the Kruskal–Wallis rank-sum test and Dunn’s test with Bonferroni corrections (*: *p* ≤ 0.05, **: *p* ≤ 0.01).

Bray–Curtis-based beta diversity showed differential geographic patterns for surface and reef-depth microbial communities. For surface waters, the western and central reefs both show more variability compared to off-reef sites (Figure 5A). In contrast, for reef-depth sites, the eastern reefs had higher beta diversity (Figure 5B). A comparison of beta diversity by protection levels showed the lowest dispersion in the most-protected reefs, but this was only significant between the medium- and high-protection sites (Figure 5C,D).

### 3.4. Microbial Community Composition

**Figure 6:**
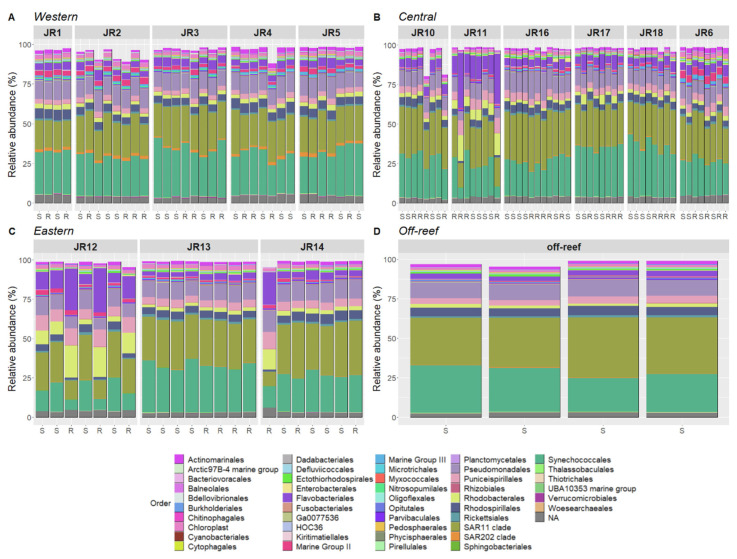
The relative abundances of the most prevalent taxonomic groups of bacteria and archaea, representing 80–95% of sequences per sample for Western (**A**), Central (**B**), Eastern (**C**) and off-reef (**D**) sites. The samples are organized according to site and geographic location. The taxa are identified to Order. The samples’ depths are indicated on the x-axis where S is surface (<1 m) and R is reef depth (6–14 m).

Bacterial and archaeal composition of reef seawater included Synechococcales (~25% relative abundance), SAR11 clade (~25%) and Pseudomonadales (~10%). Moreover, Actinomarinales, Flavobacteriales, Marine Group III archaea, Puniceispirillales, Rhodobacterales, Rhodospirillales, and Rickettsiales were consistently represented in all the samples (Figure 6). The western reefs showed elevated Nitrosopumilales, Marine Group II archaea, and SAR202 clade bacteria compared to all other geographic locations, in both surface and reef-depth communities.

Several reefs showed strong variation in taxonomic composition (Figure 6). Indeed, reefs JR11, JR12, and JR14 were characterized by higher proportions of Flavobacteriales, Rhodobacterales, and Puniceispirillales and were associated with lower proportions of Synechococcales, the SAR11 clade, and Rhodospirillales at reef depth.

The R package “corncob” was used to identify ASVs that were significantly differentially abundant between geographic locations. This analysis identified 104 ASVs that were significantly differentially abundant when the western reefs were compared to both the central and eastern reefs (Supplementary Figure S3). The most significantly enriched ASVs (coefficient from 3.75 to 5) include the taxa Nitrosococcales, Polyangiales, Rhodospirillales, Nitrosopumilales, the SAR11 clade, and Planctomycetales. The most significantly depleted ASVs (coefficient from −3.75 to −2.5) include the taxa Enterobacterales, Desulfovibrionales, Cyanobacteriales, and Flavobacteriales. Note that some taxa were both positively and negatively enriched such as Flavobacteriales (three ASV-enriched and five ASV-depleted), Rhodobacterales (two ASV-enriched, one ASV-depleted) or SAR11 clade (two ASV-enriched, three ASV-depleted). Also, Nitrosopumillales, SAR202, and Marine Group II ASVs were enriched in the western reefs (Figure 6).

Furthermore, this corncob analysis identified 93 differentially abundant ASVs in abnormal reefs (JR11, JR12, and JR14) in comparison to all the other reefs (Supplementary Figure S4). The most significantly enriched ASVs (coefficient from 1.5 to 3) on abnormal reefs included the taxa Cytophagales, Pseudomonadales, Rhodobacterales, Deinococcales, Flavobacteriales, Caenarcaniphilales, and Sphingobacteriales. The most significantly depleted ASVs (coefficient from −6 to −3) included the taxa Planctomycetales, Parvibaculales, HOC36, the Arctic97B-4 marine group, Rhodospirillales, and Opitutales.

### 3.5. Relationship between Reef Properties and Microbial Communities

**Figure 7:**
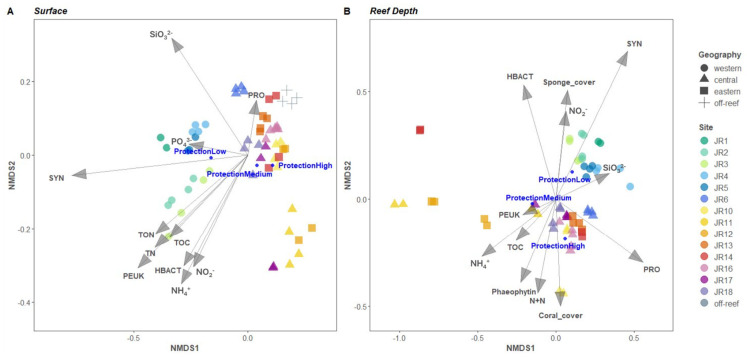
Nonmetric multidimensional scaling (nMDS) analysis of seawater bacterial and archaeal communities. The vector overlays represent multiple correlations between the ordination axes, environmental variables, and category of reef protection level (displayed as dark blue circles). Clustering was conducted using group average linkages on Bray–Curtis dissimilarity indices computed from relative abundances of 16S rRNA gene amplicons grouped into an amplicon sequence variant (ASV) from surface (**A**) and reef depth (**B**) samples.

An nMDS ordination of the microbial community structure for combined surface and reef-depth microbial communities indicated that the western reefs were more closely grouped and distinct from the central and eastern reefs and that reefs JR11 and JR12 were distinct compared to the other reefs (Figure 7). Off-reef sites also appear closely grouped on Figure 7A but closer to the central and eastern reefs. A PERMANOVA examined the influence of environmental properties on the microbial community composition (Table 3). At both depths, the microbial community composition was significantly influenced by the sites’ geographic location (R^2^ = 42% and Pr ≤ 0.001 at surface level, R^2^ = 29% and Pr ≤ 0.001 at reef depth) as well as by their protection level (R^2^ = 26% and Pr ≤ 0.001 at surface level, R^2^ = 18% and Pr ≤ 0.001 at reef depth). Chlorophyll a, algal cover, as well as fish abundance, species richness, and biomass did not significantly influence microbial communities’ structure across JR reefs (Pr > 0.05). Surface seawater microbial communities were impacted by the following (in order of significance): ammonium concentrations, abundances of *Synechococcus*, picoeukaryotes and unpigmented picoplankton, silicate concentrations, TON and total nitrogen concentrations (R^2^ range 8–68%, Pr ≤ 0.001), followed by TOC concentrations, abundances of *Prochlorococcus*, and nitrite and phosphate concentrations (R^2^ range of 5.5–6.6%, Pr ≤ 0.01). All these parameters influenced western-reef microbial composition except for *Prochlorococcus* which had a major influence on the central (especially JR6) and eastern reefs (especially JR13) as well as on off-reef sites (Figure 7A). For the reef-depth samples (Figure 7B), the western reefs’ microbial communities were mostly correlated with *Synechococcus*, *Prochlorococcus*, and unpigmented picoplankton abundances (R^2^ range of 11–24%, Pr ≤ 0.001) followed by silicate concentrations (R^2^ = 7.7%, Pr ≤ 0.01), and then by sponge cover and nitrite concentrations (R^2^ range 4.7–7.7%, Pr ≤ 0.05). The central and eastern reefs’ microbial communities were correlated with *Prochlorococcus* abundances and ammonium concentrations (R^2^ range 14–23%, Pr ≤ 0.001), phaeophytin concentrations (R^2^ = 8.1%, Pr ≤ 0.01), followed by concentrations of nitrate + nitrite and TOC, coral cover, and finally picoeukaryote abundances (R^2^ range 5–5.6%, Pr ≤ 0.05). Microbial communities from the abnormal reefs JR11, JR12, and JR14 were particularly influenced by ammonium (R^2^ = 14%, Pr ≤ 0.001) and TOC concentrations (R^2^ = 5.4%, Pr ≤ 0.05).

## 4. Discussion

This study presents the most comprehensive survey of the microbial communities as well as nutrient conditions associated with Jardines de la Reina coral reefs. We show that the reefs had generally low nutrient concentrations and are dominated by oligotrophic-type microbes. Alpha and beta diversity of the microbial communities showed variability between the reef’s sites, with relationships tied to reef geography and protection levels, as well as environmental and reef benthic features. We identify several abnormal reefs whose microbial and nutrient patterns differed considerably from the other reefs. These data provide a timestamp of microbiology in a relatively pristine coral reef system that is useful for understanding a remote, well-protected Caribbean reef ecosystem as well as for comparisons during expected future reef changes.

### 4.1. Oligotrophic-Type Nutrients and Microbial Communities

Across all reef sites, we observed generally low and consistent concentrations of macronutrients and oligotrophic microbial taxa. The only previous study of JR reef nutrients was of two forereefs sampled in 2015, and here, we report similar mean TOC and TON concentrations but higher yet still low concentrations of phosphate and inorganic nitrogen [22]. *Prochlorococcus* was on average about half that of 2015 measurements (4.4 × 10^4^ cells mL^−1^ in 2017, 10 × 10^4^ cells mL^−1^ in 2015) and with a somewhat higher concentration of *Synechococcus* (3.5 × 10^4^ cells mL^−1^ in 2017, 2 × 10^4^ cells mL^−1^ in 2015) [22]. Heterotrophic bacteria decreased about 4-fold (1.8 × 106 cells mL^−1^ in 2015; 0.39 × 10^6^ cells mL^−1^ in 2017) between the 2015 study and this study [22]. This decrease in *Prochlorococcus* and heterotrophic bacteria abundances is notable and could be related to the seasonal differences (winter in 2015, fall in 2017) or the higher sampling coverage of reefs in 2017.

Microbial communities were dominated by oligotrophic taxa including SAR11, SAR86, and *Prochlorococcus* which made up 30–60% of the reef water microbial communities, except for three samples belonging to the JR11, JR12, and JR14 sites where these taxa made up to 15–23% of the community (Figure S2). These types of bacteria are common members of oligotrophic reef environments [11,40] and generally require low levels of nutrients and dissolved organic matter and have small genomes which are streamlined for success in low-nutrient environments [41,42].

Despite the dominance of reef water communities by these oligotrophic cells, alpha and beta diversity of the communities were overall much higher in the present study compared to the two forereef sites examined in 2015. The former study [22] reported 300–350 microbial taxa at the reefs, while the present study reports a range of 250 to over 500 taxa, and here, we show elevated beta diversity in the microbial communities between sites. The 2015 study covered a limited geographic area in the most-protected MPA zone, while here, we explored more sites throughout the MPA and outside. The data analysis methods were comparable but could also play a role in these differences.

While JR reefs are highly oligotrophic, this study also demonstrated relationships between benthic reef cover and microbial communities. Coral and sponge cover significantly influenced microbial composition at reef depth. Connections between microbial and benthic communities were experimentally demonstrated by Nelson et al. [15] and Weber et al. [17]. Prior work has shown that benthic organisms can recruit specific microorganisms [15,17], and field-based investigations on JR reefs were one of the first field studies to document this trend [12]. Further, metabolomics investigations from these same reefs also showed evidence of benthic signatures in the reef metabolome [17], which has direct interactions with the reef microbial community.

Connections between the 2015 and 2017 studies demonstrate consistency in reef water microbial dynamics. However, it is important to note that the two studies represent point sampling events in time; and seasons, storms, and recent marine heat waves, among other factors, can significantly influence microbial communities and their dynamics (e.g., [43]). Thus, further sampling and investigation of these reef sites are necessary to form a more robust understanding of connections between the reef water microorganisms and influencing factors for the Jardines de la Reina reefs.

### 4.2. Geographical and Reef Protection Trends

The microbial communities varied according to geography and historic protection categories. The results pertaining to reef protection should be interpreted cautiously, as they were based on historic protection levels with no updated knowledge of recent enforcement. Microbial community alpha diversity was highest in the most-protected reefs, and these same reefs had the lowest beta diversity compared to the less-protected reef sites. Microbial community richness is often related to ecosystem and organismal health in a variety of systems [44]. A study of Micronesian coral reefs identified high alpha diversity in seawater microbial communities that were collected from reefs with high coral cover [11]. Given the high coral cover and fish species richness in the protected areas of JR, it is not surprising that these microbial communities exhibit hallmark traits associated with healthy ecosystems. Indeed, evidence of the impact of protection on reef microbial communities can be observed at a larger scale as seen in the Weber et al. [22] study which compared preserved Cuban reefs to anthropized reefs of the Florida Keys. The authors discovered distinct microbial signatures between reef systems. While the Florida Keys showed signs of microbialization, as evidenced by a shift from oligotrophic and highly diverse ecosystems towards more eutrophic and less-diverse ecosystems [45], JR reefs showed signs of an oligotrophic system with a preserved dependence of the reef community on primary production by picocyanobacteria.

*Synechococcus* abundances were consistently the lowest in the central reefs as well as reefs with the highest level of protection. *Synechococcus* are typically associated with coastal reef environments and tend to thrive in environments with higher nutrient concentrations compared to the more oligotrophic environments favoring *Prochlorococcus*. A classic example is the high *Synechococcus* distribution in Kaneohe Bay, Hawaii, which diminishes in concentration near the edge of the bay, with oceanic water containing lower organic nitrogen concentrations and higher light levels being dominated by *Prochlorococcus* [46,47]. In this study, the highest *Synechococcus* abundances were associated with the eastern reefs and could be related to the slightly higher ammonium concentrations in the benthic waters within this region. Despite all reefs being sampled on the JR sites near the offshore environment, the microbial community exhibited geographic trends that suggest influence from the hydrodynamic regime. The western reefs show distinct microbial taxonomic composition. They host more heterotrophic bacteria, and are enriched in Nitrosopumilales, SAR202 clade, and Nitrosococcaless. Additionally, JR6 and JR12 showed higher abundance of Marine Group II archaea, and JR17 and JR12 both show higher TON concentrations than other reefs of the same geographic category, a characteristic shared by the western reefs. The common feature of reefs JR6, JR12, and JR17 is that they all are located on the western side of a channel linking the Caribbean Sea with the Ana Maria gulf (spanning the inner sea between the island of Cuba and the JR archipelago). Moreover, unlike the eastern and central reefs, the western reefs are not separated from the Ana Maria gulf by islands, thus allowing gulf waters to run freely. The southern Cuban coast is flushed from east to west with oligotrophic water carried by the Caribbean current [48]. Thus, the similarities between the eastern reefs and off-reef sites, as well as the singularities of some specific reefs and the western reefs, are likely due to the impact of the Caribbean current and the presence of channels communicating with the Ana Maria gulf. Indeed, this current brings oligotrophic waters to the eastern end of the JR archipelago before carrying more eutrophic waters flowing out of the Ana Maria gulf towards the western end of the archipelago. Such results demonstrate the influence of hydrography and topography on microbial community structure. This important influence of hydrodynamic regime on the JR archipelago’s ecosystem was previously inferred by Hernandez-Fernandez et al. [28] and Marzo-Pérez et al. [49] following their work on JR’s coralline and cryptofaunal nematode communities, respectively.

Differences in beta diversity between protection levels might be partly, if not totally, due to geographical differences. Indeed, reefs with medium-protection level show high beta diversity. Yet, the eastern reefs are more influenced by Caribbean oligotrophic waters while the western reefs show evidence of the more eutrophic Ana Maria gulf waters. Furthermore, reefs with high protection show lower beta diversity, but this category gathers only three reefs (against five and six for medium- and low-protection categories) all at less than 40 km apart in the central area of the JR archipelago.

### 4.3. Abnormal Reefs: Distinguished by Microbial Community

Several reefs harbored significantly different microbial communities compared to the other reefs in the dataset. These ‘abnormal’ sites included JR11, JR12, and JR14 and were all located in the eastern side of the archipelago. Further, abnormalities in microbial community composition were primarily in benthic waters. Trends included enrichment in copiotrophic taxa including Rhodobacterales and Flavobacteriales, with specifically enriched taxa varying by reef site, associated with a depletion in oligotrophs in the SAR11 and SAR86 (ASV14) clades. These sites may be especially ideal for continued monitoring of the microbial community, for early signs of reef disruption. Further, analyses of microbial functions, such as through analyses of microbial functional genes or gene transcripts, could provide additional insights into the contribution of these unique microbial communities to the reef’s biogeochemistry and health.

## 5. Conclusions

This study demonstrated that the JR reefs are consistently oligotrophic in nutrient concentrations and are generally dominated by oligotrophic-type microorganisms. Indeed, JR reefs showed overall homogeneity in physicochemical properties, low nutrient concentrations, and consistent microbial taxonomic composition, primarily dominated by cyanobacteria and SAR11. The establishment of a MPA in 1996 seems to have succeeded in preserving the oligotrophic paradigm of the JR reef ecosystem. Several sites showed distinct microbial communities, potentially indicating disturbance and requiring further research. Reef geography was strongly linked to the microbial community, likely related to the complex hydrodynamic regimes of the archipelago. As coral reefs continue to undergo unprecedented changes worldwide, continued monitoring and research on this Caribbean ‘Crown Jewel’ reef system is important for understanding connections involving microorganisms within biodiverse reef ecosystems.

## Figures and Tables

**Table 1:** Sampled sites and grouping categories (protection level established in 1996 [23,24]).

Site	Date	Latitude (N)	Longitude (E)	Maximum Sampled Depth (m)	Geographic Location	Protection Level
JR1	4 November 2017	21.4800	−79.6544	10	Western	Low
JR2a	20 November 2017	21.3059	−79.5926	9	Western	Low
JR2b	20 November 2017	21.3033	−79.5911	9	Western	Low
JR3a	19 November 2017	21.1838	−79.5456	8	Western	Low
JR3b	19 November 2017	21.1927	−79.5504	9	Western	Low
JR4a	5 November 2017	21.0640	−79.4261	10	Western	Low
JR4b	5 November 2017	21.0638	−79.4273	14	Western	Low
JR5a	6 November 2017	20.9672	−79.1975	12	Western	Medium
JR5b	6 November 2017	20.9654	−79.2046	12	Western	Medium
JR6a	7 November 2017	20.8435	−79.0099	12	Central	Medium
JR6b	7 November 2017	20.8439	−79.0217	12	Central	Medium
JR10a	12 November 2017	20.7764	−78.9167	10	Central	High
JR10b	12 November 2017	20.7750	−78.9189	10	Central	High
JR11a	11 November 2017	20.6729	−78.7531	9	Central	Medium
JR11b	11 November 2017	20.6800	−78.7546	12	Central	Medium
JR12a	10 November 2017	20.6269	−78.5946	12	Eastern	Medium
JR12b	10 November 2017	20.6241	−78.5888	14	Eastern	Medium
JR13a	8 November 2017	20.5791	−78.4492	9	Eastern	Low
JR13b	8 November 2017	20.5791	−78.4427	6	Eastern	Low
JR14a	9 November 2017	20.5131	−78.3864	11	Eastern	Low
JR14b	9 November 2017	20.5065	−78.3811	6	Eastern	Low
JR16a	14 November 2017	20.7539	−78.8373	12	Central	High
JR16b	14 November 2017	20.7568	−78.8400	12	Central	High
JR16c	15 November 2017	20.7595	−78.8456	14	Central	High
JR17a	16 November 2017	20.7167	−78.7960	14	Central	High
JR17b	16 November 2017	20.7125	−78.7877	14	Central	High
JR18a	17 November 2017	20.8548	−79.0485	14	Central	Medium
JR18b	17 November 2017	20.8495	−79.0373	12	Central	Medium
OR1	9 November 2017	20.5134	−78.4131	1	Off-reef	Off-reef
OR2	11 November 2017	20.7467	−78.8784	1	Off-reef	Off-reef

**Table 2:** Overview of nutrient concentrations and cell abundances for JR coral reef, with surface and benthic water values combined (d.l. = detection limit, SD = standard deviation).

Environmental Variable	Range (Min–Max)	Mean (SD)
TOC (µM)	49.0–158.9	72.9 (15.0)
TON (µM)	0.07–12.0	4.5 (2.4)
PO_4_^3−^ (µM)	0.13–0.38	0.18 (0.05)
NO_3_^−^ + NO_2_^−^ (µM)	d.l.–0.68	0.19 (0.12)
NO_2_^−^ (µM)	d.l.–0.08	0.04 (0.02)
NH_4_^+^ (µM)	0.03–1.6	0.26 (1.3)
Silicate (µM)	0.91–1.9	1.5 (0.23)
*Prochlorococcus* (cells mL^−1^)	14,920–94,795	44,127 (13,904)
*Synechococcus* (cells mL^−1^)	5990–84,304	34,117 (16,915)
Picoeukaryotes (cells mL^−1^)	464–15,412	1047 (1995)
Nonpigmented cells (cells mL^−1^)	237,178–703,575	397,424 (76,634)

**Table 3:** PERMANOVA results of the effect of environmental properties on the microbial community structure in JR seawater. The parameters in bold have a major influence on community structure (0.047 < R^2^ < 0.773 and Pr ≤ 0.05). (Df = degree of freedom, Pr = probability).

Surface (<1 m)			
Property	Df	R^2^	Pr
**Site**	14	0.773	**0.001**
**Geography**	3	0.420	**0.001**
**Protection**	3	0.258	**0.001**
**Total organic carbon**	1	0.066	**0.002**
**Total nitrogen**	1	0.081	**0.001**
**Phosphate**	1	0.055	**0.005**
Nitrate + nitrite	1	0.027	0.141
**Silicate**	1	0.103	**0.001**
**Nitrite**	1	0.062	**0.002**
**Ammonium**	1	0.675	**0.001**
**Total organic nitrogen**	1	0.087	**0.001**
***Prochlorococcus***	1	0.063	**0.002**
***Synechococcus***	1	0.243	**0.001**
**Picoeukaryotes**	1	0.105	**0.001**
**Heterotrophic bacteria**	1	0.101	**0.001**
Reef depth (6–14 m)			
Property	Df	R^2^	Pr
**Site**	14	0.705	**0.001**
**Geography**	3	0.290	**0.001**
**Protection**	3	0.180	**0.001**
**Total organic carbon**	1	0.054	**0.033**
Total nitrogen	1	0.014	0.520
Phosphate	1	0.012	0.632
**Nitrate + nitrite**	1	0.056	**0.019**
**Silicate**	1	0.077	**0.006**
**Nitrite**	1	0.047	**0.038**
**Ammonium**	1	0.138	**0.001**
Total organic nitrogen	1	0.016	0.463
Chlorophyll *a*	1	0.037	0.067
**Phaeophytin**	1	0.081	**0.007**
***Prochlorococcus***	1	0.230	**0.001**
***Synechococcus***	1	0.240	**0.001**
**Picoeukaryotes**	1	0.050	**0.026**
**Heterotrophic bacteria**	1	0.114	**0.001**
Fish abundance	1	0.047	0.159
Fish species diversity	1	0.022	0.537
Fish biomass	1	0.036	0.272
Algae cover	1	0.032	0.129
**Coral cover**	1	0.054	**0.024**
**Sponge cover**	1	0.077	**0.011**

## Data Availability

Raw sequences are available in the NCBI Short Read Archive (SRA) under project number PRJNA1010761.

## Supplementary Materials

The following supporting information can be downloaded at: https://www.mdpi.com/article/10.3390/microorganisms12091822/s1, Figure S1: Microbial alpha diversity indices of samples organized by depth type (Surface: <1 m, Reef depth: 6–14 m); Figure S2: Relative abundances of the dominant oligotrophic taxa of bacteria and archaea present within surface and reef depth waters; Figure S3: Differentially abundant ASVs identified in western reefs when compared to both eastern and central reefs using the R package ‘corncob’ (v 0.3.1); Figure S4: Differentially abundant ASVs identified in abnormal reefs JR11, JR12 and JR14 using the R package ‘corncob’ (v 0.3.1).
