# Supplementary material for: Coral Reef Water Microbial Communities of Jardines de la Reina, Cuba
Source: Microorganisms. 2024 Sep 3;12(9):1822. doi: 10.3390/microorganisms12091822 (PMC11433942; doi:10.3390/microorganisms12091822)
Supplement: Supplementary file 1 [file microorganisms-12-01822-s001.zip › microorganisms-3165442-supplementary.pdf]

Supplementary material

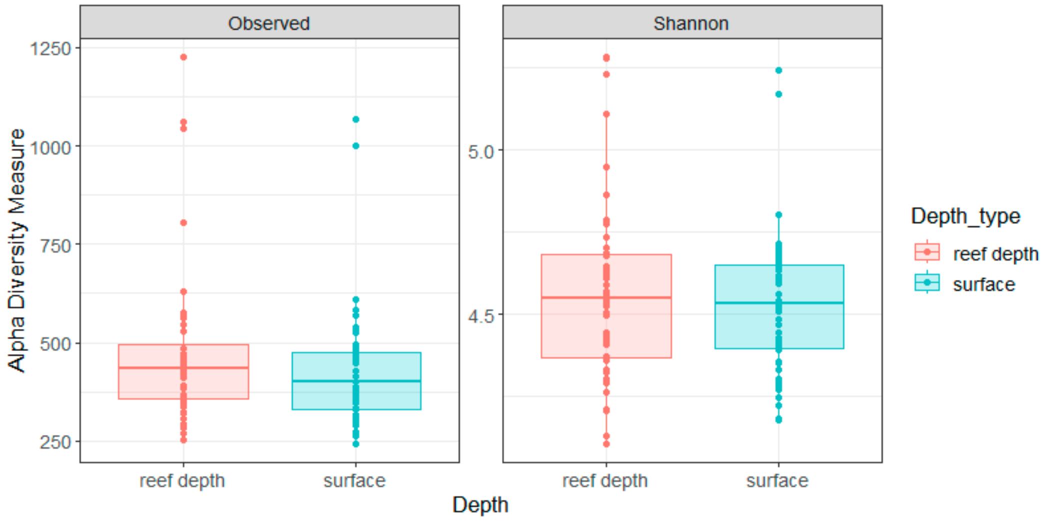

Figure S1: Microbial alpha diversity indices of samples organized by depth type (Surface : <1 m, Reef depth : 6-14 m). The horizontal thick bar across the boxplot represents the median value and the whiskers extend to values at 1.5 times the interquartile range (delimited by the box).

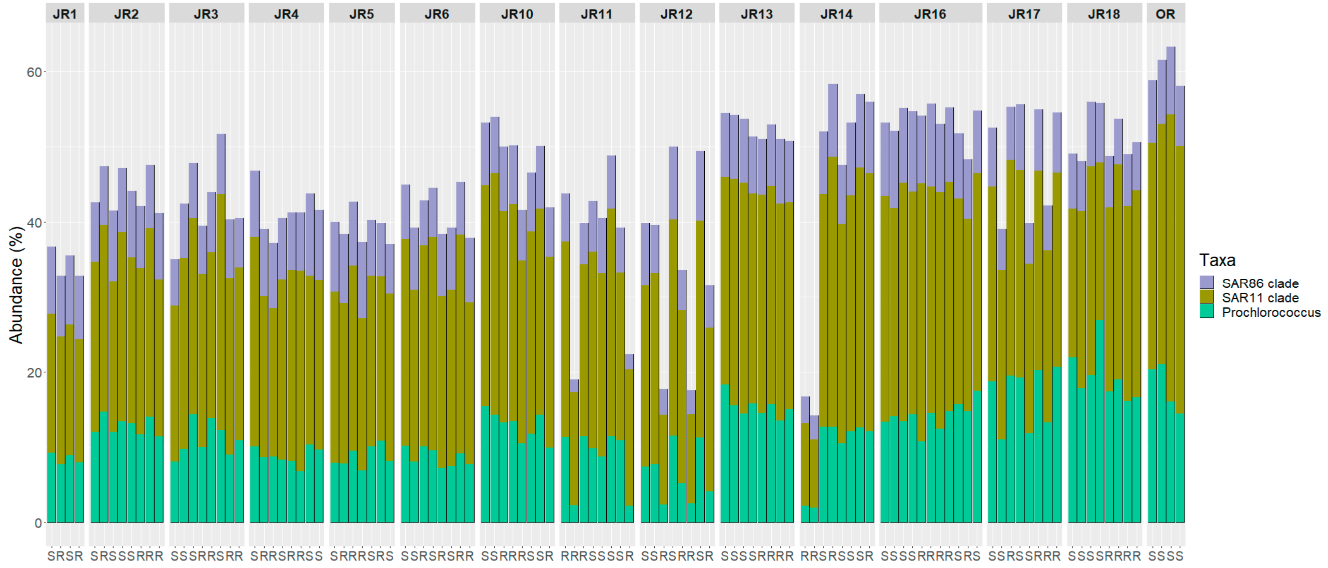

Figure S2: Relative abundances of the dominant oligotrophic taxa of bacteria and archaea present within surface and reef depth waters. Samples' depths are indicated on the x axis where R stands for Reef depth (6-14m) and S for Surface (<1m).

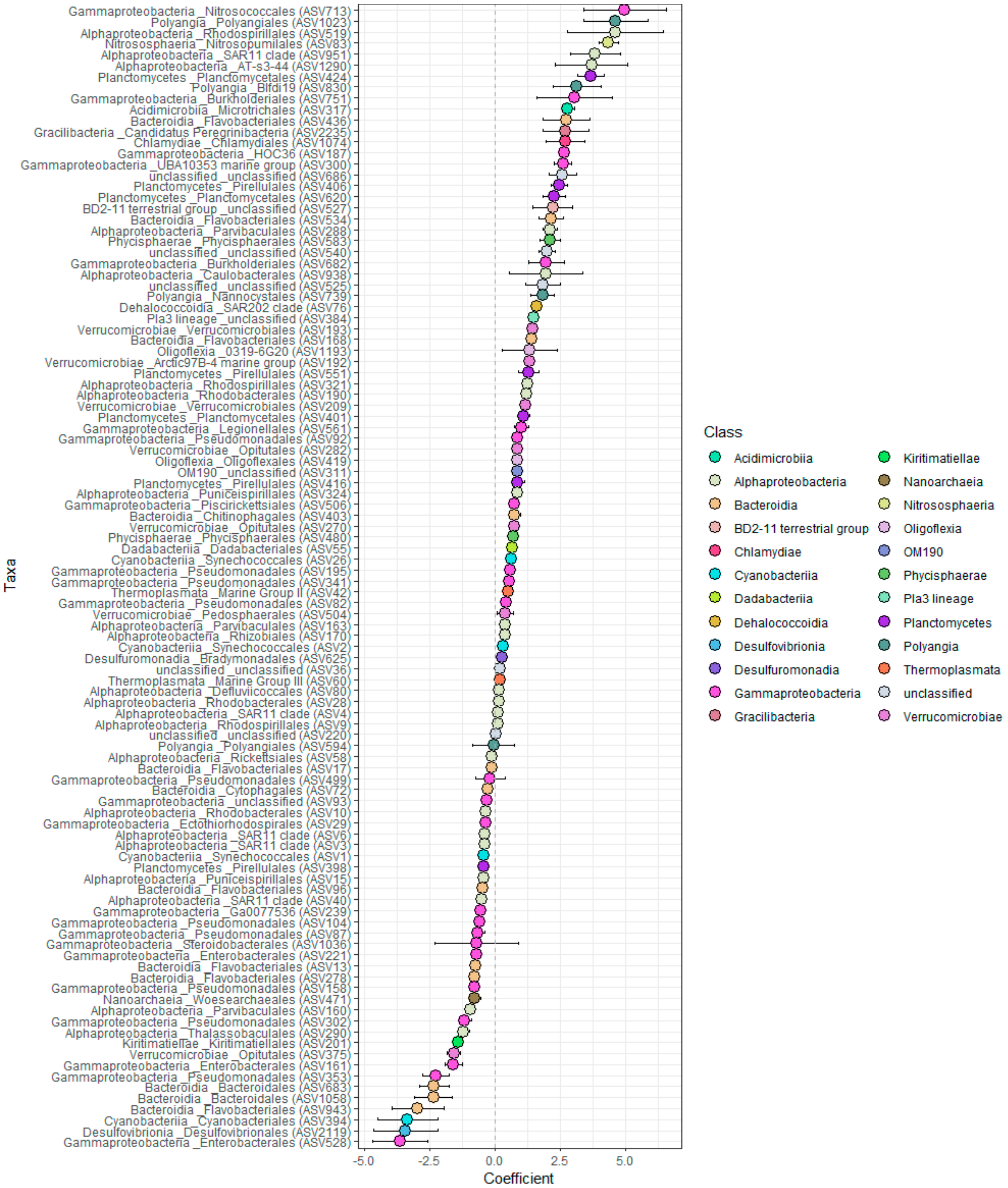

Figure S3; Differentially abundant ASVs identified in western reefs when compared to both eastern and central reefs using the R package 'corncob' (v 0.3.1). The coefficient indicates the change in ASV abundance compared to both eastern and central reefs (outlier reefs and off-reef sites excluded). ASVs are named in order by class and order taxonomic levels.

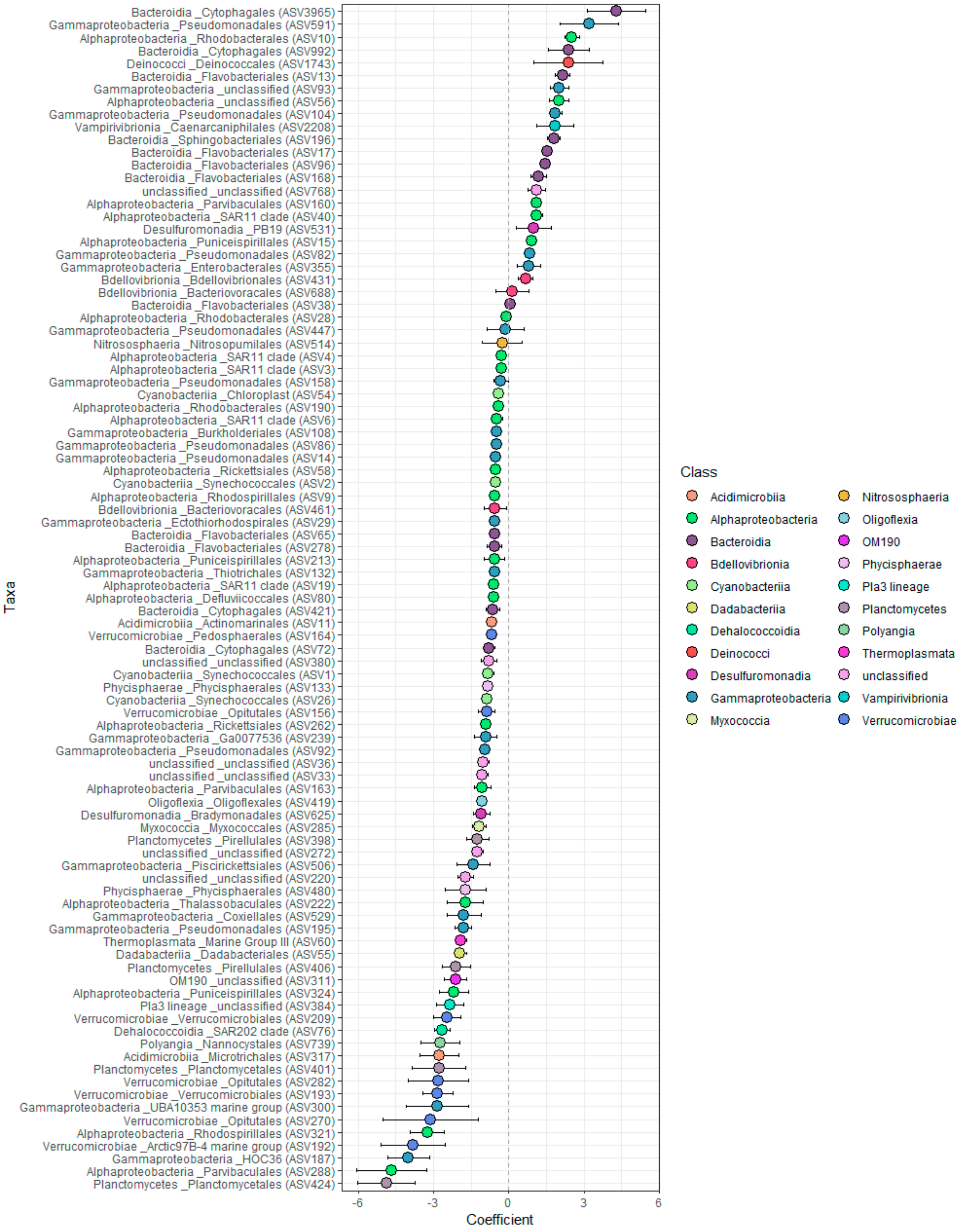

Figure S4: Differentially abundant ASVs identified in abnormal reefs JR11, JR12 and JR14 using the R package 'corncob' (v 0.3.1). The coefficient indicates the change in ASV abundance compared to all other reefs (surface and off-reef samples excluded). ASVs are named in order by class and order taxonomic levels.
